# Mapping the Strong‐to‐Weak Coupling Crossover in Polymer‐Film Microcavity Lasers

**DOI:** 10.1002/nap2.70214

**Published:** 2026-07-22

**Authors:** Denis A. Sannikov, Nailya M. Urazova, Maksim D. Kolker, Grigorij D. Ivanov, Anton D. Putintsev, Aleksandr V. Averchenko, Daria Khmelevskaia, Liliya T. Sahharova, Nikita S. Shlapakov, Ioannis Paschos, Pavlos G. Savvidis, Valentine P. Ananikov, Pavlos G. Lagoudakis

**Affiliations:** ^1^ Hybrid Photonics Laboratory Skolkovo Institute of Science and Technology Territory of Innovation Center Skolkovo Moscow Russia; ^2^ Zelinsky Institute of Organic Chemistry Russian Academy of Sciences Moscow Russia; ^3^ Westlake University Hangzhou Zhejiang China

**Keywords:** conjugated polymers, exciton‐polaritons, light‐matter coupling, MeLPPP, photon lasing, polariton lasing, tunable laser

## Abstract

Organic semiconductors are particularly attractive for polaritonics due to their large exciton binding energies and oscillator strengths. Among them, the ladder‐type conjugated polymer poly(paraphenylene) is distinguished by its rigid backbone, narrow exciton linewidth, high photoluminescence quantum yield, and enhanced photostability, making it an excellent candidate for organic polariton devices. Although polariton lasing has been reported in various organic systems, systematic studies of the transition from polariton lasing to conventional photon lasing within a single, well‐controlled material platform remain limited. Here, we present planar organic microcavities incorporating MeLPPP as the active medium, in which continuous tuning of the effective cavity length within a single device enables us to map the strong‐to‐weak coupling transition across five distinct cavity‐mode orders. We demonstrate an approximately eighteen‐fold increase in the lasing threshold when crossing from polariton to photon lasing. We further establish a quantitative framework in which the spectral dependence of the threshold governs a universal V‐shaped blueshift of the emission energy across both coupling regimes. Finally, we show that vibron‐mediated exciton relaxation, previously identified in the strong‐coupling limit, persists across the crossover: lasing‐threshold minima track the vibron resonances throughout the coupling transition.

## Graphical abstract

The transition from polariton lasing to conventional photon lasing is mapped continuously within a single MeLPPP‐filled open microcavity. The threshold rises approximately eighteen‐fold across the crossover, whereas the saturation‐driven V‐shaped blueshift and dominance of vibron‐mediated relaxation persist across both regimes, separating polariton‐specific signatures from universal gain‐driven behavior.

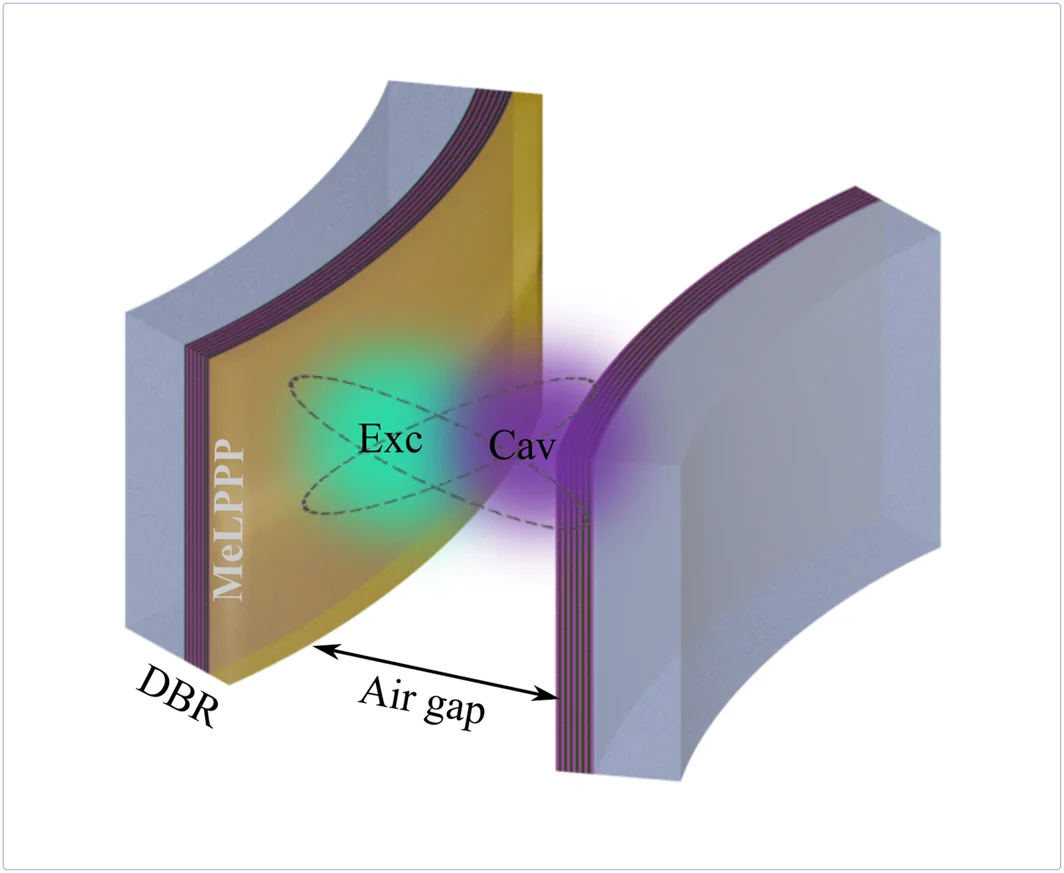

## 1 Introduction

Strong coupling between confined photonic modes and semiconductor excitonic transitions in optical microcavities gives rise to hybrid light–matter quasiparticles known as exciton–polaritons [1, 2]. Organic semiconductors are particularly well suited for realizing room‐temperature polariton phenomena [3, 4, 5] owing to the large binding energies and oscillator strengths of Frenkel excitons. To date, polariton condensates have been demonstrated in diverse organic systems, such as organic crystals [6], oligofluorenes, fluorescent proteins [7], molecular dyes [8, 9, 10], and conjugated polymers [11].

Among these candidates, conjugated polymers have attracted considerable interest for both fundamental polaritonics and optoelectronic device applications such as light‐emitting diodes [12], polymer lasers [13], photovoltaic cells [14], field‐effect transistors [15], and batteries [16, 17]. They combine the electronic functionality of semiconductors with the mechanical flexibility and solution processability of plastics, enabling the fabrication of large‐area, optical‐quality thin films at low cost. The π‐electron delocalization over chromophores in conjugated polymers enhances their transition dipole moment, giving rise to large Rabi splitting values (> 500 meV) under strong coupling conditions even in simple planar microcavities [18].

Within this family, the methyl‐substituted ladder‐type polymer poly(paraphenylene) (MeLPPP) is particularly notable. Its rigid, rod‐like backbone, stabilized by methylene bridges, produces narrow exciton linewidth with distinct vibronic structure, a small Stokes shift, and a high PL quantum yield up to 30% [19, 20], along with the enhanced photostability [21]. Microcavities incorporating MeLPPP have enabled the realization of exciton–polariton condensates [22] and advanced polariton‐based functionalities, such as tunable lasing [23, 24], multimode lasing [25], ultrafast all‐optical transistors [26, 27], cascadable logic gates [28] leveraging subTHz temporal dynamics [29], high degree of second‐order coherence [30] of polariton condensates, and quantum simulations in engineered photonic lattices [31, 32].

The transition between conventional photon lasing, based on population inversion, and polariton lasing, driven by bosonic stimulation of exciton–polariton scattering, has been studied in organic systems by different methods including changing cavity length [33], increasing molecular density in active layer (superabsorption) [34], and exploiting polarization‐dependent birefringence in aligned perylene diimide films [35]. Nevertheless, no results have been reported for a continuous range of cavity lengths within a single, photostable material platform with a well‐resolved vibronic structure. Understanding the crossover between these regimes is essential for distinguishing polariton‐specific signatures from conventional lasing and for optimizing device design.

Vibron‐assisted relaxation has been established as an important mechanism in strongly coupled organic microcavities [36, 37], and the deliberate exploitation of vibronic resonances under resonant pumping has been used to reduce condensation thresholds by an order of magnitude [26]. Whether this channel persists within the strong‐coupling regime, and how the threshold modulation evolves as the system is tuned towards the weak‐coupling regime, has not, however, been systematically addressed.

In this work, we present a straightforward fabrication procedure for planar, strongly coupled organic microcavities incorporating the conjugated ladder‐type polymer MeLPPP as an active medium capable of supporting polariton lasing. By systematically varying the effective cavity length within a single device, we track the transition from strong to weak coupling through the polariton condensation threshold, emission linewidth, and energy shift as functions of nonresonant optical pump fluence. The single‐device mapping, spanning five cavity mode orders that straddle the Savona criterion $\hbar \Omega_{R}>\Gamma_{\text{exc}}+\Gamma_{\text{cav}}$ [38], provides the continuous detuning trajectory. On this trajectory we derive and test a quantitative link between the spectral dependence of the lasing threshold and the spectral dependence of the nonlinear energy shift at threshold. Building on the saturation‐driven blueshift mechanism of Ref. [39], in which the emission energy is pulled by Pauli‐blocking‐induced saturation of the molecular transitions, we show that the magnitude of the shift is set by the lasing threshold itself, which is in turn governed by the gain spectrum of the active medium. Since the gain spectrum is centered on the amplified spontaneous emission (ASE) peak, the predicted blueshift follows a parabolic (V‐shaped) dependence as a function of the spectral offset $\Delta E=E_{\text{ASE}}-E_{k_{0}}$ between the ASE maximum and the lasing mode. The framework also predicts that the V‐shape acquires a nonzero baseline value at ΔE=0 in the strong‐coupling regime, arising from a quenching of the Rabi splitting under saturation, and that this baseline vanishes once strong coupling is lost, providing, in principle, a coupling‐regime‐dependent diagnostic in addition to the universal V‐shape itself. Within the same dataset, the vibron‐mediated‐relaxation picture of Refs. [26, 36, 37] is tested within the strong‐coupling regime. We find that the lasing‐threshold minima track the $V_{2}$ and $V_{3}$ vibron resonances for the strongly coupled mode orders and that this modulation smoothly weakens as the coupling strength is reduced towards the weak‐coupling limit. Throughout, we employ nonresonant, ultrafast (250 fs, 400 nm) excitation, which populates the molecular density uniformly and therefore interrogates the coupling‐regime dependence without the spectral selectivity inherent in resonant vibronic pumping. The combined result is an approximately eighteen‐fold increase in the lasing threshold between the polariton and photon lasing regimes at their respective optimal exciton‐photon detunings relative to the material gain maximum, and a decomposition of the energy‐shift data that distinguishes universal gain‐driven behavior from polariton‐specific signatures.

## 2 Experimental Results

We synthesize Me‐LPPP precipitate following published procedures [40, 41, 42, 43, 44, 45], and characterize the resulting material by measuring the Raman spectrum of a 180‐nm thin film, spin‐coated onto SiO_2_/Si substrate (see Figure 1a) with the chemical structure of the polymer depicted in Figure 1b. We observe nearly identical spectrum as reported previously [26, 46], with the slightly varying relative intensities of V1‐, V2‐, and V3 vibron bands, for details on the synthesis procedure see Supporting Information S1: Section S1. The normalized absorption spectrum of the MeLPPP thin film is shown in Figure 1c depicted with blue solid line. Two well‐resolved peaks in the absorption spectrum are assigned to optical transitions from the ground singlet state S_0_ to sublevels of the first excited singlet state S_1_: the purely electronic (0–0) transition at 449 nm (2.76 eV) and the first vibronic (0–1) sideband at 421 nm (2.94 eV). Their Stokes‐shifted counterparts in the fluorescence spectrum shown with red solid line in Figure 1c are observed at 463 nm (2.68 eV) and 493 nm (2.51 eV), respectively. The material's ability to manifest optical gain was characterized by measuring ASE along a stripe excitation profile (see Supporting Information S1: Section S2 for setup schematics). ASE spectrum of a 180‐nm thin film is shown with black solid line in Figure 1c. The gain peak, identified by a nonlinear rise in emission intensity at a threshold incident pump fluence of 4.9 μJ ${\text{cm}}^{-2}$, is centered at 487.25 nm (2.545 eV) and approximately coincides with (0–1) vibrational transition, suggesting a four‐level lasing scheme [8].

**FIGURE 1:**
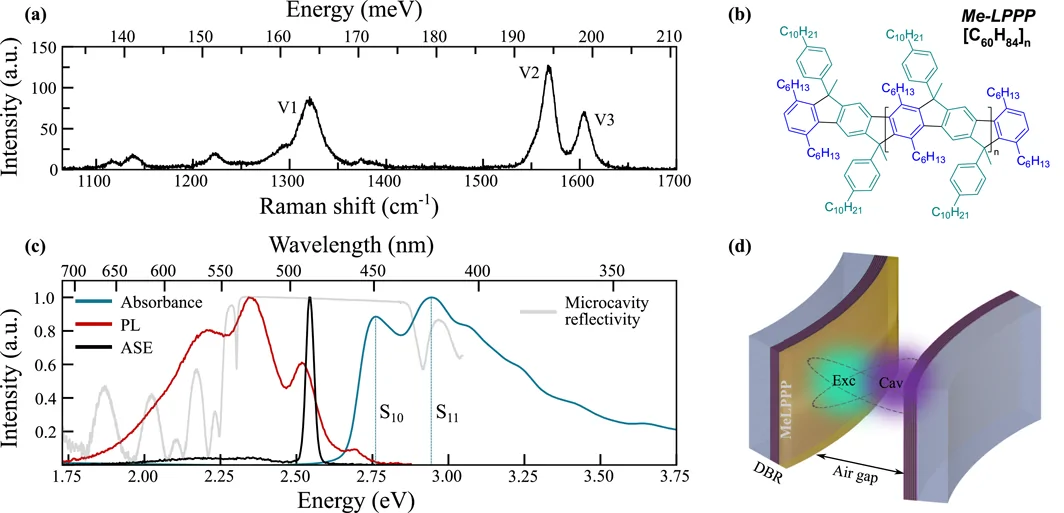
Material characterization. (a) Raman spectrum of methyl‐substituted ladder‐type polymer poly(paraphenylene) (MeLPPP) reference, 180‐nm, noncavity thin film with three highlighted molecular vibrational modes V1, V2, and V3. (b) Chemical structure of the polymer chain. (c) Normalized absorption (blue line) with two highlighted excitonic transitions $S_{10}$ and $S_{11}$, fluorescence (red line), amplified spontaneous emission 487.25 nm/2.545 eV (black line) spectra of the corresponding film. Gray line shows the microcavity reflectivity spectrum. (d) Schematic structure of the asymmetric open microcavity: a MeLPPP polymer film is spin‐coated onto the bottom DBR (Ta_2_O_5_/SiO_2_, Ta_2_O_5_‐terminated), and a second DBR is positioned above the polymer layer with a variable air gap, forming an asymmetric open‐cavity geometry whose effective cavity length is tuned by lateral translation of the sample.

We fabricate microcavities by spin‐coating a 180‐nm‐thick MeLPPP film onto a high‐reflectivity distributed Bragg reflector (DBR), consisting of 12.5 pairs of alternating Ta_2_O_5_ (n≈2.14)/SiO_2_ (n≈1.46) quarter‐wavelength thick layers, with the high‐index Ta_2_O_5_ layer terminating at the polymer interface to maximize field overlap with the active medium (see Methods Section 4 for details). A second DBR is then positioned above the polymer layer using a point‐squeeze mounting apparatus [47], creating an asymmetric open‐cavity geometry with an air gap between the polymer surface and the top mirror. This configuration yields a Q−factor of ≈2000 within the stop‐band center. Numerical calculations confirm that one of the electric‐field antinodes is located at the polymer layer for the cavity modes of interest (for details see Supporting Information S1: Section S3). A one‐time compression procedure then induces the curvature in the top and bottom DBRs, forming a convex‐shaped microcavity with an optical cavity thickness increasing radially from a minimum at the compression point. The effective cavity length is therefore tuned by translating the sample laterally, which displaces the pump spot to regions of different thickness relative to the compression point. The sample geometry is illustrated schematically in Figure 1d.

To distinguish the strong‐ and weak‐coupling regimes quantitatively, we adopt the standard criterion of Savona et al. [38]: strong coupling requires the Rabi splitting $\hbar \Omega_{R}$ to exceed the sum of the exciton and cavity linewidths, $\hbar \Omega_{R}>\Gamma_{\text{exc}}+\Gamma_{\text{cav}}$. From the absorption spectrum of a 180‐nm MeLPPP reference film, we extract $\Gamma_{\text{exc}}=92.0\pm 2.4$ meV (FWHM); and from the below‐threshold cavity dispersions, we extract $\Gamma_{\text{cav}}=7.86\pm 0.28$ meV (FWHM), yielding a criterion threshold of $\Gamma_{\text{exc}}+\Gamma_{\text{cav}}=99.9\pm 2.4$ meV. In what follows, we characterize the lasing behavior at three representative cavity‐mode orders—M=3 (strong coupling, $\hbar \Omega_{R}\;\approx 170$ meV > the criterion), M=8 (in the immediate vicinity of the Savona criterion, classified as an intermediate regime), and M=17 (weak coupling, $\hbar \Omega_{R}\to 0$).

Under normal‐incidence, 250‐fs, nonresonant optical excitation spectrally tuned at 400 nm, the cavity is optically pumped from the polymer‐DBR side (bottom) to maximize absorption in the active layer, and photoluminescence is collected in transmission through the air‐gap side (top). We perform either a multipulse integrated imaging of PL emission for the below‐lasing‐threshold regime or a single‐shot PL imaging of the above‐lasing‐threshold emission in k‐space as a function of excitation density recorded in transmission configuration (see Section 4) for a set of cavity lengths. This asymmetric excitation–collection geometry has been shown to influence mode characteristics in open cavities [48]. Across all measurements, we tune the effective cavity length in a wide range from 700 nm to 4.2 μm. For length values between 700 and 1250 nm, that correspond to the strongly coupled regime, we observe a nonlinear increase in the PL intensity integrated over ±5° around normal incidence, concomitant narrowing of the emission full‐width at half maximum (FWHM) line width, and the step‐like blueshift of the emission energy, as evidenced by blue, red, and black axes in Figure 2a, respectively. This set of data is acquired for 731‐nm effective cavity thickness and the third‐order cavity photon mode (M=3) and is chosen to demonstrate an indicative behavior of the studied regime. These three characteristics constitute the key signatures of polariton lasing [39, 49], providing clear evidence for its occurrence.

**FIGURE 2:**
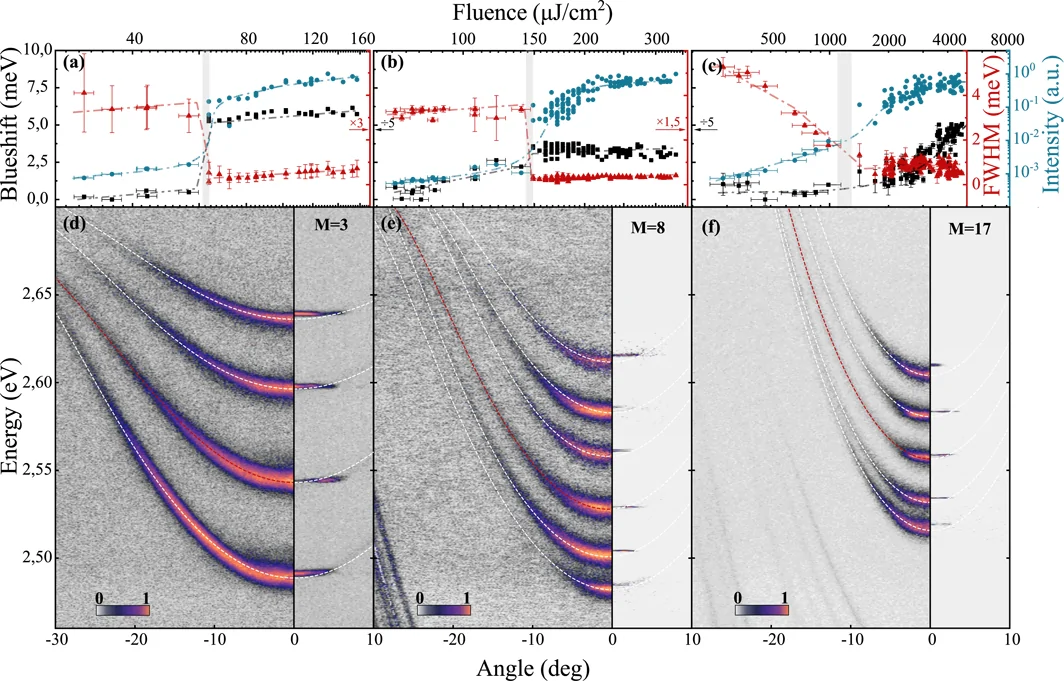
Hallmarks of polariton and photon lasing. (a) The polariton PL intensity (blue circles), linewidth (red triangles), and energy shift (black squares) as a function of pumping fluence in a strongly coupled regime (order M=3, effective cavity length $L_{\text{eff}}=731\;nm$) with the polariton lasing threshold indicated with the vertical gray stripe, $P_{\text{th}}^{\text{pol}}=62.6\;\mu J\;{cm}^{-2}$. (b) Same as (a) for the intermediate coupling regime (order M=8, $L_{\text{eff}}=1962\;nm$) with the lasing threshold $P_{\text{th}}=145\;\mu J\;{cm}^{-2}$. (c) Same as (a) for the weakly coupled regime (order M=17, $L_{\text{eff}}=4122\;nm$) with the photon lasing threshold $P_{\text{th}}^{\text{ph}}=1100\;\mu J\;{cm}^{-2}$. In all three panels, a step‐like blueshift of the emission energy is observed at threshold. Dashed curves in panels (a)–(c) are guides to the eye. Energy shift values are multiplied by a factor of 5 (b), (c). Linewidth values are divided by a factor of 3 (a), 1.5 (b). (d)–(f), Dispersion images of PL below (left subpanels) and above (right subpanels) the lasing threshold for M=3 (d), M=8 (e), and M=17 (f), each shown at several different cavity lengths corresponding to different exciton–photon detuning conditions. White dashed lines in panels (d)–(f) represent the best‐fit results of a coupled‐oscillators model. The red dashed lines in panels (d)–(f) indicate the modes whose lasing hallmarks are plotted in the corresponding panels (a)–(c) above. The color scale is normalized to the maximum intensity of the above‐threshold dispersion images.

Figure 2d shows the dispersion images of the below (left subpanel) and above (right subpanel) the lasing threshold PL for four distinct detuning conditions Δ= −58, −107, −174 and −235 meV (*M* = 3). Above the polariton lasing threshold $P_{\text{th}}^{\text{pol}}$, the k‐space PL distribution collapses towards $k_{\|}=0\pm 5\;{}^{\circ}$, indicating the onset of coherent macroscopic occupation at the ground, lowest energy state. Together with the gradual broadening of the polariton lasing linewidth above the threshold, these hallmark behaviors evidence the presence of the strong light–matter coupling enabling the emergence of new polariton eigenstates inside the system. To quantify the interaction strength of the system, namely the Rabi‐splitting energy constant, we apply a coupled‐oscillators model with damping and fit the experimentally observed PL dispersion images, as indicated by white dashed lines of lower and upper polariton branches (LPB and UPB) in Figure 2d. The Hamiltonian of the system reads as follows:

(1) $$\hat{H}=\left(\begin{array}{cc} E_{\text{cav}|k}-i\gamma_{\mathrm{cav}} & V\\ V & E_{\mathrm{exc}}-i\gamma_{\mathrm{exc}} \end{array}\right)$$

here, $E_{\text{cav}|k}$ is the eigenenergy of the cavity photon mode, $E_{\mathrm{exc}}$ is the $S_{10}$ exciton energy, $\gamma_{\mathrm{cav}}=\Gamma_{\mathrm{cav}}/2$, $\gamma_{\mathrm{exc}}=\Gamma_{\mathrm{exc}}/2$ and V is the interaction strength of the photon–exciton coupling for the $S_{00}\to S_{10}$ optical transition. Rabi splitting $\hbar \Omega_{R}$ is related to interaction strength V by $\hbar \Omega_{R}=\sqrt{4V^{2}-{\left(\gamma_{\mathrm{cav}}-\gamma_{\mathrm{exc}}\right)}^{2}}$. In our case $|\gamma_{\mathrm{cav}}-\gamma_{\mathrm{exc}}|=42$ meV and the square root that defines Rabi splitting value remains purely real for all analyzed dispersions. We restrict the coupled‐oscillator description to the $S_{10}$ exciton because the $S_{11}$ transition at 2.94 eV falls outside the high‐reflectivity stop‐band of our DBRs, where the cavity Q‐factor drops from ≈2000 at the stop‐band center to ≈70 (see the reduced reflectivity of the cavity near the $S_{11}$ line of the absorption spectrum in Figure 1c); the cavity finesse is therefore insufficient for $S_{11}$ to participate in the observed polariton physics; and coupling to $S_{11}$ is effectively absent within the measurement range of this work.

We now turn to the intermediate and weak‐coupling regimes announced at the outset. At the intermediate effective cavity length $L_{\text{eff}}=1962$ nm (M=8), the pump‐fluence dependence and dispersion images are shown in Figure 2b,e, respectively. Compared with the strongly coupled M=3 case, the lasing threshold rises by roughly a factor of four, the step‐like blueshift at threshold persists but is reduced to less than half of its M=3 magnitude, and the dispersion deflection, though still identifiable in the coupled‐oscillator fit (white dashed lines in Figure 2e), is markedly weaker. These intermediate‐regime signatures are consistent with the Savona criterion placing M=8 in the immediate vicinity of the strong‐coupling boundary rather than firmly inside it. At the largest cavity length investigated ($L_{\text{eff}}=4122$ nm, M=17, Figure 2c,f), the lasing threshold increases by more than an order of magnitude relative to M=3, and the dispersion acquires a bare‐cavity parabolic shape without visible anticrossing, indicating that the system has crossed into the weak‐coupling regime where lasing occurs via conventional population inversion rather than bosonic stimulation.

Extending the Savona‐criterion analysis to all five measured mode orders (Figure 3), M=3 and M=5 exhibit $\hbar \Omega_{R}=140$–170 meV and clearly satisfy the strong‐coupling criterion; M=8 and M=9 yield $\hbar \Omega_{R}=100$–110 meV, in the immediate vicinity of the threshold, and are therefore classified as an intermediate regime; and M=17 shows $\hbar \Omega_{R}$ approaching zero together with a narrow, nearly‐parabolic dispersion, consistent with the weak‐coupling regime. As an independent indicator of the same crossover, we extract the effective mass of each measured dispersion (Figure 3, top panel), which gradually decreases from $∼8\times 10^{-5}\;m_{e}$ ($m_{e}$ is an electron mass) at small cavity lengths to $∼2\times 10^{-5}\;m_{e}$ at large cavity lengths, being a signature of the increasing photonic character of the lower branch as the coupling weakens.

**FIGURE 3:**
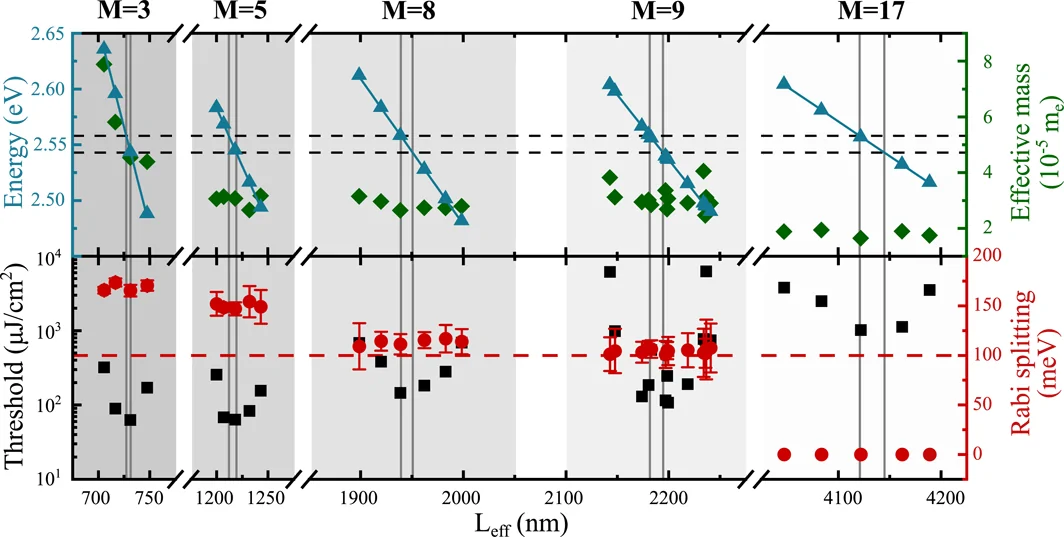
Cavity length tuning dependencies. Lasing threshold fluence (black squares markers, left axis of the bottom panel), Rabi splitting constant $\hbar \Omega_{R}$ (red circle markers, right axis of the bottom panel), emission energy from $k_{\|}=0$ of either the LPB or the cavity photon mode (blue triangle markers, left axis of the top panel), and polariton effective mass (green diamond markers, right axis of the top panel) are plotted as functions of the effective cavity length $L_{\text{eff}}$ for five mode orders, M=3,5,8,9,17. According to the Savona criterion $\hbar \Omega_{R}>\Gamma_{\text{exc}}+\Gamma_{\text{cav}}=99.9\pm 2.4$ meV (red horizontal dashed line in the bottom panel) [38], modes M=3 and M=5 ($\hbar \Omega_{R}\approx 140$–170 meV) correspond to the strong‐coupling regime, modes M=8 and M=9 ($\hbar \Omega_{R}\approx 100$–110 meV) lie in the immediate vicinity of the criterion and are classified as intermediate, whereas M=17 $\left(\hbar \Omega_{R}\to 0\right)$ corresponds to the weak‐coupling regime. The effective mass decreases gradually from $∼8\times 10^{-5}\;m_{e}$ at small cavity lengths to $∼2\times 10^{-5}\;m_{e}$ at large cavity lengths, providing a model‐independent signature of the strong‐to‐weak coupling transition. Horizontal‐dashed (upper panel) and vertical‐solid (bottom panel) lines indicate spectral positions of V2 and V3 vibron resonances with respect to the $S_{10}$ exciton.

A natural question is whether the nonlinear energy shift at threshold carries coupling‐regime‐specific information beyond what the lasing thresholds themselves reveal. Following the framework of Yagafarov et al. [39], the saturation‐induced blueshift in organic microcavities is linear in the saturation parameter ξ, which under uniform nonresonant pumping is proportional to the threshold fluence $P_{\text{th}}$. Because $P_{\text{th}}\propto 1/\sigma_{\text{gain}}\left(E_{k_{0}}\right)$ and the gain cross‐section $\sigma_{\text{gain}}\left(E\right)$ is smooth with a maximum at $E_{\text{ASE}}$, a second‐order Taylor expansion yields a parabolic threshold dependence: $P_{\text{th}}\propto \left[1+{\left(\Delta E/w\right)}^{2}\right]$, where $\Delta E=E_{\text{ASE}}-E_{k_{0}}$ and w is a characteristic gain width (see Supporting Information S1: Section S4 for a detailed analysis). This framework therefore predicts that the total energy shift should follow

(2) $$E_{\text{lasing}}-E_{k_{0}}=A_{\text{sat}}\cdot {\left(\Delta E/w\right)}^{2}+A\cdot \Delta E+E_{0}$$

with a symmetric, parabolic V‐shape in ΔE, an antisymmetric component A⋅ΔE arising from any gain‐induced frequency pulling, and a baseline $E_{0}$ set by coupling‐regime‐specific contributions (notably Rabi quenching, which should contribute in the strong‐coupling limit and vanish in the weak‐coupling limit). An independent estimate of the gain‐pulling contribution yields less than 1 meV across the entire spectral range, owing to the narrow cavity linewidths ($\Delta \omega_{c}∼1.5$–10 meV) relative to the broad ASE bandwidth (∼40 meV); we therefore expect A≈0 and a symmetric V‐shape across all coupling regimes.

Figure 4 tests this prediction directly, showing the measured energy shifts at $\approx 2\times P_{\text{th}}$ relative to the linear (below‐$P_{\text{th}}$) emission at $k_{\|}=0\pm 5\;{}^{\circ}$ for three representative cavity orders: M=5 (strong coupling, yellow diamonds), M=9 (intermediate, black squares), and M=17 (weak coupling, green triangles). The data follow the predicted parabolic V‐shape across all three regimes, with RMS residuals of 0.8–1.1 meV and no detectable antisymmetric component (consistent with A=0 within experimental precision). The baseline $E_{0}$, which the saturation framework of Ref. [39] associates with Rabi‐splitting quenching and which should therefore grow with the coupling strength in the strong‐coupling regime and vanish in the weak‐coupling limit, is extracted from the fit for each cavity order: M=5 (strong coupling, $\hbar \Omega_{0}\approx 140$ meV) yields $E_{0}=+0.3$ meV, whereas M=9 and M=17 yield near‐zero baselines. The ordering of these values is consistent with the theoretical expectation, but the fitted $E_{0}$ for M=5 is comparable to the RMS residual of the fit itself, so within the present experimental precision the data do not allow a statistically meaningful verification of the baseline as a coupling‐regime diagnostic. Disentangling this contribution from experimental scatter would require either measurements at deeper strong coupling (where a larger Rabi splitting should amplify the Rabi‐quenching contribution) or a reduction in the per‐point energy‐shift uncertainty; both are natural directions for future work. The universality of the V‐shape itself across all coupling regimes nevertheless confirms, in agreement with Ref. [39], that the energy‐shift magnitude is governed primarily by the threshold‐driven saturation parameter and does not by itself distinguish polariton from photon lasing.

**FIGURE 4:**
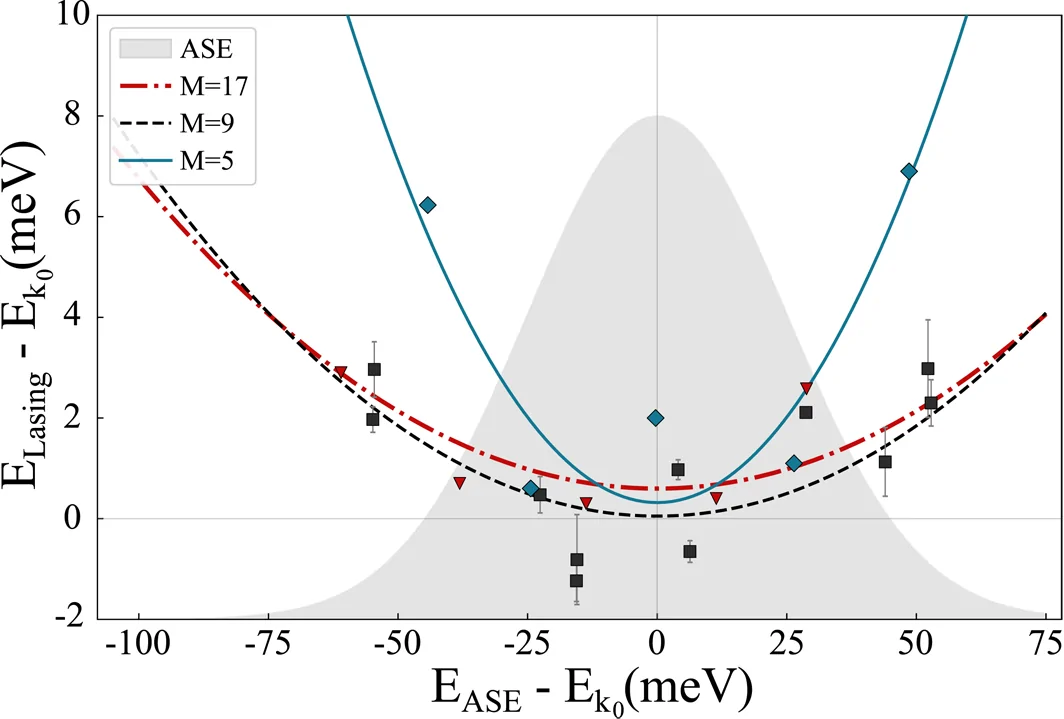
Energy shifts of lasing emission. Energy shift of the nonlinear PL emission at $\approx 2\times P_{\text{th}}$ with respect to the linear, below‐$P_{\text{th}}$ emission from $k_{0}\equiv k_{\|}=0\pm 5\;{}^{\circ}$, for cavity orders M=5 (yellow diamonds, strong coupling), M=9 (black squares, intermediate coupling), and M=17 (green triangles, weak coupling) as a function of energy offset between the ASE maximum, $E_{\text{ASE}}=2.545\;eV$, and the ground‐state energy of the dispersion, $E_{k_{0}}$. Solid and dashed curves show the best fits by the parabolic model $E_{\text{lasing}}-E_{k_{0}}=A_{\text{sat}}\cdot {\left(\Delta E/w\right)}^{2}+E_{0}$. The gray shaded area shows the normalized ASE spectrum of a 180‐nm thin MeLPPP film.

Having established the three representative regimes, we now examine the full detuning dependence across all five measured mode orders (Figure 3). In the strong‐coupling regime (M=3 and M=5), the interaction strength $\hbar \Omega_{R}$ for a specific cavity mode monotonically decreases with increasing detuning, as theoretically predicted (Figure 3, bottom panel).

The lowest threshold fluence for polariton lasing is achieved when the exciton‐to‐polariton transition matches the energy of the most pronounced vibronic resonances, V2 and V3, see vertical and horizontal dashed lines in Figure 3. The defining role of interactions between Frenkel excitons, polaritons, and molecular vibrational modes on the overall dynamics of the light–matter coupled system was previously experimentally demonstrated and theoretically considered in other systems [36, 37, 50, 51]. Additionally, the minimal observed lasing threshold in the weakly coupled regime (M=17 in Figure 3) exceeds the lowest polariton lasing threshold at the optimal detuning (corresponding to M=3 and M=5 in Figure 3) by approximately a factor of 18, whereas at suboptimal detunings the weak‐to‐strong coupling threshold ratio exceeds one order of magnitude.

## 3 Discussion

The experimental results demonstrate the decisive role of cavity length and coupling strength in governing the lasing dynamics of MeLPPP‐based organic microcavities. The observed increase in lasing threshold (approximately eighteen‐fold when comparing optimal conditions) across the strong‐to‐weak coupling transition unambiguously distinguishes polariton lasing, driven by bosonic stimulation, from conventional photon lasing that relies on population inversion. A threshold contrast of comparable magnitude between the polariton and photon lasing regimes has been reported previously in laminated fluorescent‐protein microcavities [33], where both thresholds were observed as sequential pump‐power transitions at each detuning position within the strong‐coupling regime using a wedged cavity that scans detuning on a single sample. Here, by continuously tuning the effective cavity length within a single MeLPPP device, we resolve the strong‐to‐weak coupling transition as a continuous function of detuning rather than as two endpoint observations, allowing Rabi splitting, effective mass, and lasing threshold to be tracked along the same specimen from M=3 to M=17.

Beyond the threshold comparison itself, the quantitative decomposition of the nonlinear energy shifts (Figure 4) provides a second independent signature of the coupling regime. The V‐shaped dependence of the blueshift on spectral offset from the ASE peak is universal across all three regimes, confirming the conclusion of Ref. [39] that blueshifts alone do not distinguish polariton from photon lasing. The baseline $E_{0}$ extracted from the parabolic fit is within the fit uncertainty consistent with zero in the weakly coupled regime and marginally positive ($E_{0}=+0.3$ meV) at M=5, in qualitative agreement with the Rabi‐quenching prediction of Ref. [39] but not at a statistical confidence level that would support it as a stand‐alone diagnostic. A definitive demonstration of the baseline as a coupling‐regime fingerprint requires data at deeper strong coupling and with improved per‐point precision, which we identify as a direction for future work.

In the strong coupling regime, the lasing emission exhibits characteristic features of polariton condensation, including a nonlinear increase of the polariton PL intensity, spectral blueshift, and linewidth narrowing while crossing the condensation threshold. As the system transitions into the weak coupling regime, these signatures diminish, and the emission energy converges towards the material gain maximum, reflecting the onset of photon lasing behavior. The ability to systematically tune the effective cavity length thus provides a powerful means of probing the continuous evolution between hybrid light–matter and purely photonic lasing regimes within a single material platform. Moreover, the correlation between reduced lasing threshold and vibronic resonance conditions highlights the important role of vibron‐assisted exciton relaxation in enabling efficient polariton formation and condensation in conjugated polymers, consistent with theoretical predictions [50, 51] and prior experimental observations in organic microcavities [26, 36, 37]. Our systematic study across multiple cavity lengths and mode orders provides additional evidence for this mechanism and extends its characterization into the intermediate and weak‐coupling regimes, where the threshold modulation at the vibronic resonances smoothly weakens as the coupling strength is reduced.

These findings reinforce MeLPPP as a highly suitable model system for exploring fundamental aspects of strong coupling and polariton dynamics in organic semiconductors. Notably, the asymmetric open‐cavity geometry employed here, with the active polymer layer on one side of the air gap, represents one possible configuration. A symmetric design with polymer layers on both cavity mirrors could potentially increase the effective oscillator strength and extend the strong‐coupling regime to larger detunings. However, such a configuration would also increase absorption losses, and the net effect on lasing threshold would depend on the balance between enhanced coupling and increased optical losses. Optimization of cavity symmetry and field distribution represents an interesting direction for future device engineering. Looking forward, engineering of cavity architectures and photonic lattice configurations could enable control of vibronic interactions and polariton dispersion, paving the way toward low‐threshold, room‐temperature polariton devices and integrated organic polaritonic circuits.

## 4 Methods

### 4.1 Sample Fabrication

The DBRs were fabricated by sputter‐depositing of 12.5 pairs of alternating Ta_2_O_5_/SiO_2_ quarter wavelength‐thick layers onto a sapphire substrate with Ta_2_O_5_ terminating layer.

To prepare the precursor solution at a concentration of 1 mM, 21 mg of MeLPPP was dissolved in 1 mL of toluene via sonication for 5 min. A 1 ${\text{cm}}^{2}$ substrate featuring a DBR was cleaned sequentially in ultrasonic bath using acetone, isopropanol and deionized water, with 1 min for each step, then dried using nitrogen. Subsequently, the substrate underwent treatment in a plasma asher for 15 min in a mixture of argon and nitrogen to enhance surface hydrophilicity.

For the deposition of MeLPPP, the precursor solution was spin‐coated onto the substrate at a speed of 5000 rpm for 1 min, with a ramp rate of 500 rpm/s. During this process, 30 μL of the precursor solution was dynamically dispensed once the desired rotational speed was achieved. Finally, a second DBR (on a separate sapphire substrate) was positioned above the MeLPPP‐coated surface using a point‐squeeze mounting apparatus. The apparatus applies localized pressure at a single point, creating an open‐cavity geometry where the air gap between the polymer surface and the top DBR increases radially from the contact point. This configuration allows continuous tuning of the effective cavity length by translating the sample laterally. No adhesive bonding or thermal treatment is used; the cavity assembly is maintained by the mechanical mounting.

An additional noncavity reference MeLPPP film was spin‐coated onto SiO_2_/Si substrate under conditions completely identical to the cavity's active layer.

The active layer thickness of 180 ± 20 nm in the cavity was estimated from profilometer (Alpha‐Step D‐600, KLA‐Tencor (USA)) measurements of a reference noncavity MeLPPP film.

### 4.2 Spectroscopy

The absorption measurements of the MeLPPP reference noncavity film were performed using a Lambda 1050 UV/Vis/NIR spectrometer (PerkinElmer).

ASE measurements of the reference film were performed by using a high energy Ti:Sapphire regenerative amplifier (Coherent Libra‐HE) providing ∼250 fs pulses at a 500 Hz repetition rate. The amplifier's output was then converted to 400 nm via second‐harmonic generation in a BBO crystal. A 50 mm cylindrical lens was used to focus the beam on the sample creating a stripe excitation profile (1790 μm × 95 μm). Stimulated emission of a reference noncavity MeLPPP film was detected from the edge of the film, in the direction of the stripe and perpendicular to the propagation direction of the incident pump beam using Ocean Optics QE PRO spectrometer.

For real‐ and momentum‐space imaging of the MeLPPP microcavity output emission, excitation optical pulses of ≈ 250 fs duration at 400 nm, generated by frequency‐doubling the output of a 500 Hz high energy Ti:Sapphire regenerative amplifier (Coherent Libra‐HE) in BBO crystal, were used. An optical beam was spectrally filtered using a long‐pass filter Semrock FF01‐750/LP‐25 and focused onto the microcavity with a Nikon Plan Apo 20 × microscopic objective, producing a Gaussian spot with a diameter of ≈ 10 μm at the $1/e^{2}$ intensity level. Photoluminescence was collected in the transmission configuration (a schematic of the experimental setup is provided in Supporting Information S1: Section S5). After passing through the collection objective (Mitutoyo Plan Apo 50 ×), which provides a ≈±36° detection window in reciprocal space, the emission was spectrally filtered (ThorLabs LP442) and coupled into the spectrometer (Princeton Instruments ProEM 1024BX). The Fourier plane of the collection objective was projected onto the entrance slit of the spectrometer using an additional conjugate lens. An additional imaging camera (Hamamatsu Orca) was configured for the convenient control of the excitation beam focus adjustment and cavity thickness tuning. White light reflectivity measurements were performed using an Ocean Optics DH‐2000 fiber‐coupled Halogen–Deuterium white light source.

### 4.3 Threshold Determination

The lasing threshold was determined by measuring the integrated emission intensity within a narrow angular range of $k_{\|}=0\pm 5{}^{\circ}$ as a function of the pump fluence. The data were plotted on a double logarithmic scale, and linear fits were applied separately to the spontaneous emission regime (below threshold) and the stimulated emission regime (above threshold). The threshold fluence value was defined as the intersection point of these two linear approximations. Each cavity configuration was measured 3–5 times, with the final threshold fluence value derived from the combined set of measurements.

## Author Contributions

**Denis A. Sannikov:** investigation, conceptualization, methodology, supervision, funding acquisition. **Nailya M. Urazova:** investigation, methodology, formal analysis, writing – original draft. **Maksim D. Kolker:** investigation, methodology, data curation, visualization, formal analysis. **Grigorij D. Ivanov:** investigation, methodology, visualization, writing – review and editing, software, formal analysis. **Anton D. Putintsev:** conceptualization, investigation, writing – original draft, writing – review and editing, validation, project administration. **Aleksandr V. Averchenko:** investigation, methodology, writing – review and editing. **Daria Khmelevskaia:** formal analysis, methodology. **Liliya T. Sahharova:** investigation, validation, methodology, resources. **Nikita S. Shlapakov:** resources, investigation, visualization, formal analysis. **Ioannis Paschos:** resources, validation. **Pavlos G. Savvidis:** resources, validation. **Valentine P. Ananikov:** resources, supervision, project administration, writing – review and editing. **Pavlos G. Lagoudakis:** resources, supervision, project administration, writing – review and editing, conceptualization.

## Funding

This work was supported by the Russian Science Foundation (RSF) (Grant 25‐72‐00149).

## Consent

Informed consent was obtained from all individuals included in this study.

## Conflicts of Interest

The authors declare no conflicts of interest.

## Supporting information


Supporting Information S1

## Data Availability

The data that support the findings of this study are available from the corresponding author upon reasonable request.
