# Supplementary material for: Mapping the Strong‐to‐Weak Coupling Crossover in Polymer‐Film Microcavity Lasers
Source: Nanophotonics. 2026 Jul 22;15(14):e70214. doi: 10.1002/nap2.70214 (PMC13642096; doi:10.1002/nap2.70214)
Supplement: Supplementary file 1 [file NAP2-15-e70214-s001.pdf]

Supplementary Information: Mapping the strong-to-weak coupling crossover in polymer-film microcavity lasers

Denis A. Sannikov[1,•], Nailya M. Urazova[1,•], Maksim D. Kolker[1,•], Grigorij D. Ivanov[1], Anton D. Putintsev[1], Aleksandr V. Averchenko[1], Daria Khmelevskaia[1], Liliya T. Sahharova[2], Nikita S. Shlapakov[2], Ioannis Paschos[3], Pavlos G. Savvidis[3], Valentine P. Ananikov[2], and Pavlos G. Lagoudakis[1,*]

[1] *Hybrid Photonics Laboratory, Skolkovo Institute of Science and Technology, Territory of Innovation Center Skolkovo, Bolshoy Boulevard 30, building 1, 121205 Moscow, Russia*

[2] *Zelinsky Institute of Organic Chemistry, Russian Academy of Sciences, Leninsky Prospect 47, 119991 Moscow, Russia*

[3] *Westlake University, Zhe Jiang Sheng, Hang Zhou Shi, Xi Hu Qu, 310024, China*

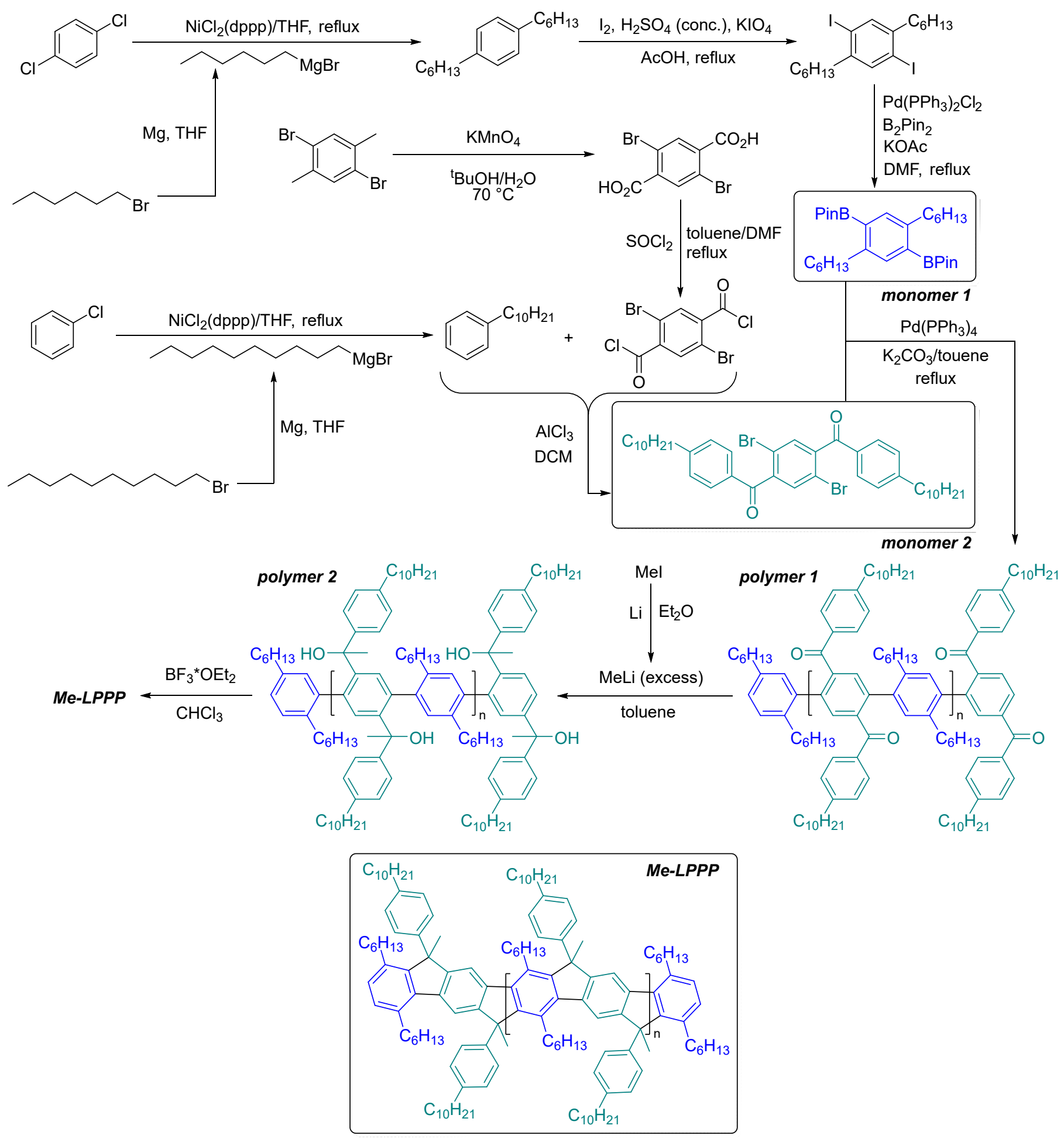

Figure S1: Step-by-step chemical process of MeLPPP polymer synthesis.

1 Synthesis procedure of MeLPPP precipitate

Monomer 1 and monomer 2 were synthesized according to published procedures (1; 2; 3). Polymer 1 was synthesized using an adapted methodology established for ladderane-type polymers (4). For this purpose, a fresh $Pd(PPh_3)_4$ catalyst was prepared (5) and used the following day. Polymer 1 was functionalized with a 100-fold excess of MeLi relative to the monomeric unit equivalent (6). A fresh MeLi solution was also prepared according to an adapted procedure (5) and used on the same day. The ^{1}H NMR spectrum registered in d8-toluene is in full agreement with the literature data, showing the appearance of characteristic signals for OH and Me groups in a 1:3 ratio in the 2-3 ppm region compared to the spectrum of the initial Polymer 1 (6). The final synthesis of Me-LPPP was performed according to a literature procedure (6). To remove oligomers, the product was dissolved in a minimal amount of toluene, and acetone was added until precipitate flocculation was observed. After allowing the mixture to stand for 2-3 hours, the polymer was separated from the mother liquor by centrifugation and decantation. The resulting Me-LPPP precipitate was washed three times with acetone and dried on a vacuum line.

2 Amplified Spontaneous Emission (ASE) measurements

Amplified spontaneous emission behavior was characterized using the experimental setup shown in Fig.S4. The polymer layer forms a planar waveguide, as its refractive index at the emission wavelength of 400 nm exceeds that of both the SiO_2/Si substrate and the surrounding air. Upon increasing the excitation fluence above a threshold of $\approx 4.9\ \mu J\ cm^{-2}$, the photoluminescence (PL) spectrum collapsed into a narrow peak centered at 487.25 nm, indicating the onset of ASE.

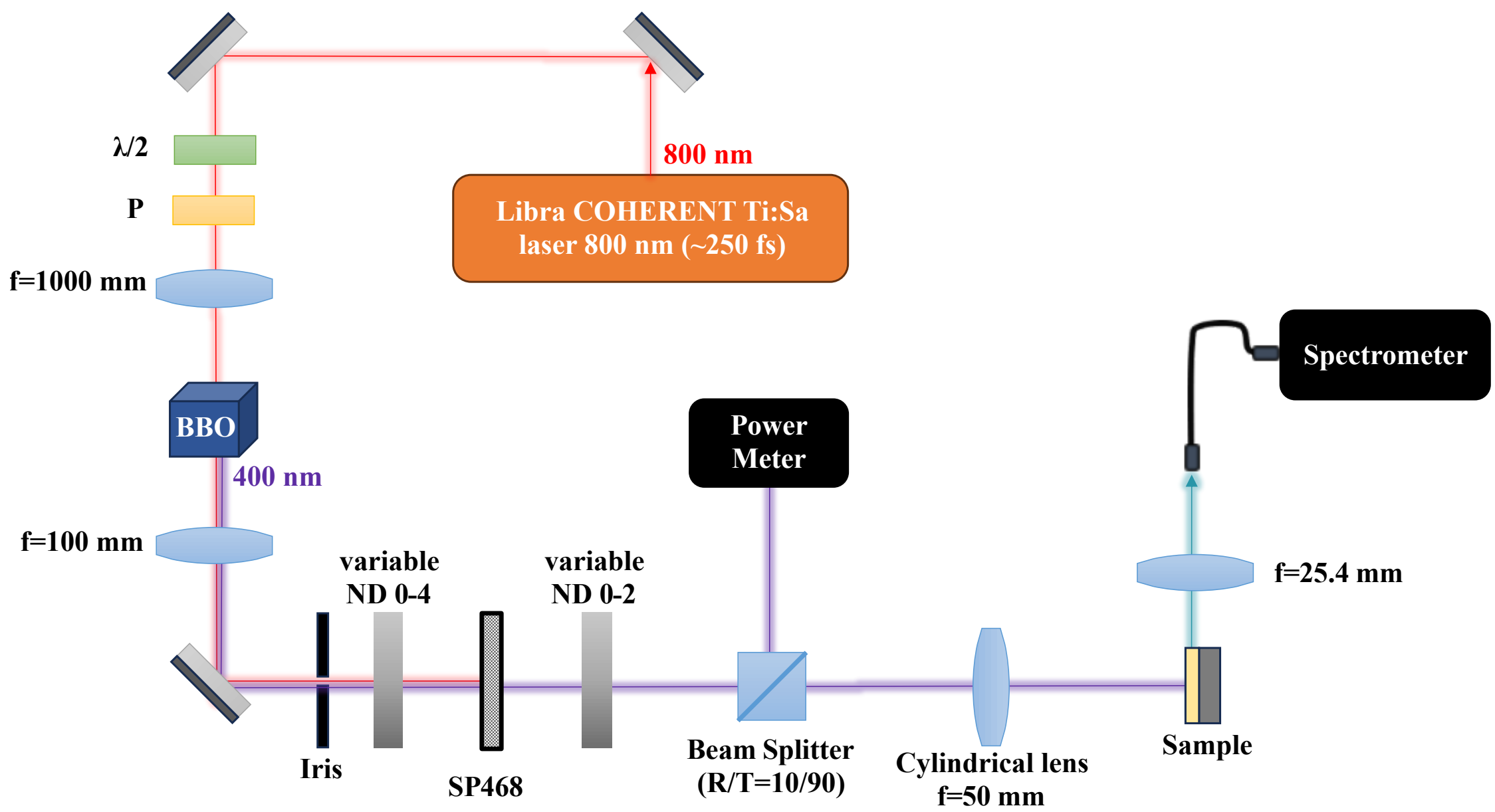

Figure S2: Schematic of the experimental setup for optical characterisation of amplified spontaneous emission of a MeLPPP thin film. Optical elements designations are provided in Fig.S5

3 Optical properties of the MeLPPP-containing DBR microcavity

Numerical calculations of the reflectance spectra and electric-field distribution were performed in COMSOL Multiphysics using the finite element method. A 2D model with periodic boundary conditions (Floquet periodicity) and two periodic input/output ports was employed. The microcavity consisted of bottom and top distributed Bragg reflectors (12.5 pairs of Ta_2O_5/SiO_2 $\lambda/4$ layers) with a MeLPPP polymer layer in between. The refractive indices were taken as n = 2.06 for the 56-nm Ta_2O_5 layer, n = 1.487 for the 85-nm SiO_2 layer, and n = 1.8 for the 180-nm MeLPPP layer.

Figure S3a shows the reflectance spectrum calculated for a microcavity with an 855-nm air gap between the MeLPPP layer and the top DBR. The pronounced mode within the stopband corresponds to the cavity mode of order M = 5, as confirmed by the electric-field distribution (Figure S3b). The out-of-plane electric-field profile shows that one of the field antinodes is located within the polymer layer (Figure S3c).

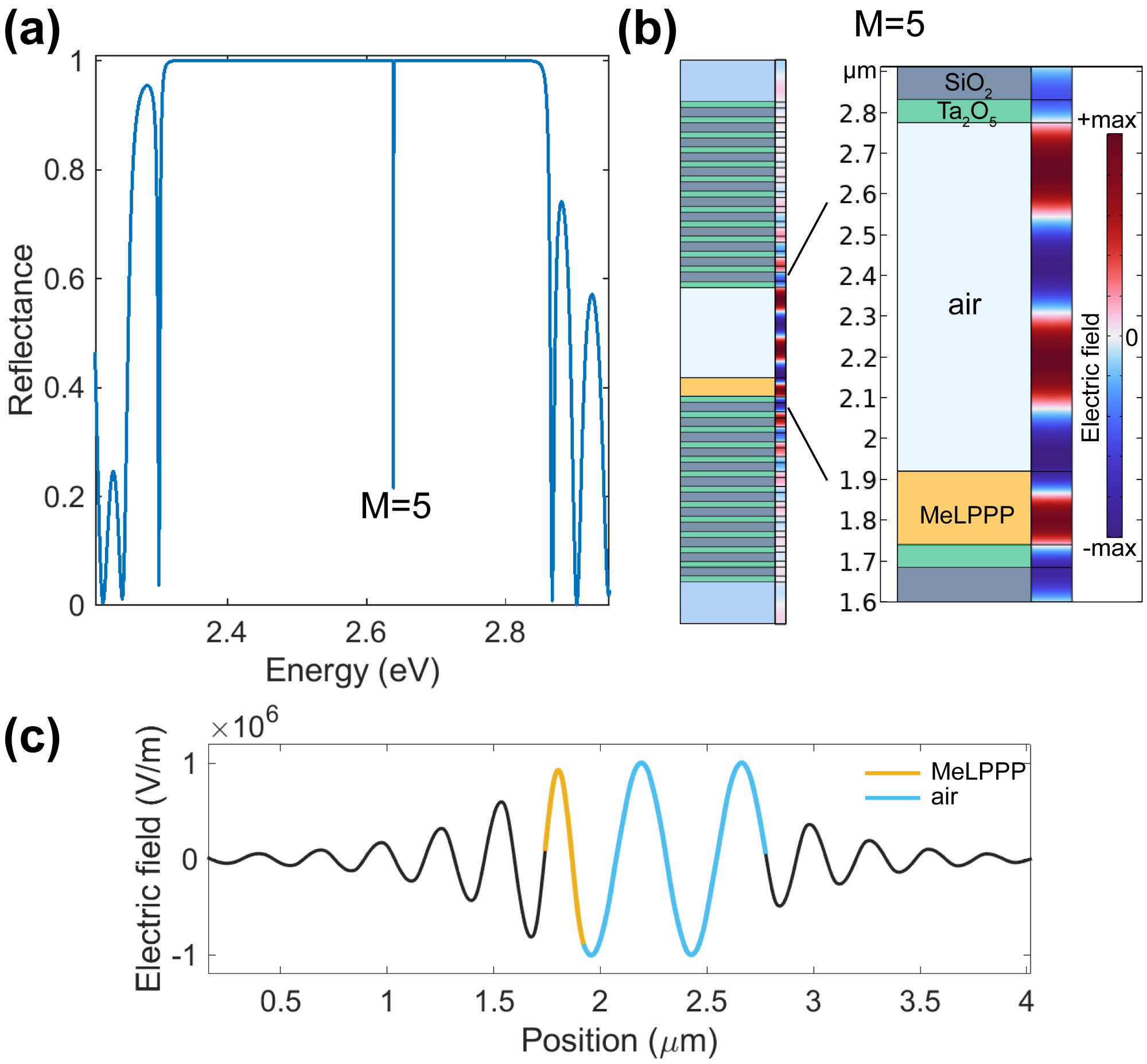

Figure S3: (a) Calculated reflectance spectrum of a microcavity with cavity mode order M = 5. (b) Schematic of the microcavity structure and spatial distribution of the out-of-plane electric field at the resonance of the M = 5 cavity mode (left panel). Magnified view of the microcavity region (right panel). (c) Spatial profile of the electric-field amplitude along the structure.

4 Derivation of the V-shape energy-shift model

The main manuscript (Eq. 2) introduces a quantitative framework that links the spectral dependence of the lasing threshold to the spectral dependence of the nonlinear energy shift via a parabolic, saturation-driven V-shape. In this section we derive that expression from the saturation picture of Yagafarov *et al.* (7), identify each of the three contributions, and quantify their relative magnitudes.

4.1 Physical mechanisms

We have conducted an extensive series of measurements with careful attention to alignment and reproducibility, and are now confident in the observed energy-shift dependence on ASE offset across all cavity mode orders studied. The total nonlinear energy shift arises from three mechanisms, which we summarise here before assembling them into the V-shape model.

Saturation-induced blueshift. Non-resonant optical pumping excites intracavity molecules, saturating their optical transitions via Pauli blocking. This produces two effects:

1. quenching of the vacuum Rabi splitting for strongly coupled molecules (Eq. 2 of Ref. (7));
2. renormalisation of the cavity mode energy via the Kramers–Kronig relation for all molecules, including the weakly coupled majority.

Both effects scale linearly with the saturation parameter

$$\xi = \frac{n_x}{n_0}, \tag{S1}$$

where n_x is the density of excited molecules and n_0 is the total molecular density. In the small-saturation limit ($\xi \ll 1$), the total blueshift from these mechanisms is given by Eq. 8 of Ref. (7):

$$\Delta E_{\mathrm{LPB}} = \frac{\xi}{2}\left[\frac{s\,\hbar\Omega_0}{\sqrt{1+s^2}} + \frac{(E_x - |\delta|)\,F[d]\,\alpha}{5\,n_{\mathrm{eff}}}\left(1 + \frac{1}{\sqrt{1+s^2}}\right)\right], \tag{S2}$$

where $s = \hbar\Omega_0/|\delta|$ is the strong-coupling parameter, $F[d]$ is the Dawson function encoding the Kramers–Kronig spectral dependence, and α is the oscillator-strength parameter. The critical point is that both terms are linear in ξ, so the blueshift magnitude is directly proportional to the saturation parameter.

Gain-induced frequency pulling. The lasing mode is pulled toward the gain maximum (the ASE peak) by an amount proportional to the ratio of the cavity linewidth to the gain linewidth. This produces an antisymmetric contribution: a redshift when the cavity mode is on the blue side of the ASE peak and a blueshift on the red side. For our system, the cavity linewidths ($\Delta\omega_c \sim 1.5$–10 meV) are much narrower than the gain bandwidth ($\Delta\omega_{\mathrm{gain}} \sim 40$ meV), making this contribution small.

Threshold-dependent saturation parameter. The key insight connecting these mechanisms to the observed spectral dependence of the energy shifts is that the saturation parameter ξ *at threshold* is not constant across the gain spectrum, but varies systematically with the spectral offset $\Delta E = E_{\mathrm{ASE}} - E_{k_0}$ from the ASE peak. This variation is governed by the lasing threshold, which we have independently measured for each data point.

4.2 Derivation of the quadratic model

We now derive the functional form of the energy-shift dependence on spectral offset. The derivation proceeds in three steps.

Step 1: Parabolic threshold dependence

At the lasing threshold, the round-trip gain must equal the round-trip losses. The net gain $g(E)$ available at cavity mode energy E is determined by the spectral overlap between the cavity resonance and the material gain profile, with the lasing threshold reached when the round-trip gain compensates the round-trip losses. The gain profile of MeLPPP, as characterised by the ASE spectrum of the bare film, is a smooth, single-peaked function of energy centred at E_{ASE}. By definition, the first derivative of any smooth function vanishes at its maximum, so the leading-order variation near E_{ASE} is quadratic — a direct consequence of the Taylor expansion to second order:

$$\begin{aligned} g(E) &= g(E_{\mathrm{ASE}}) + \left.\frac{\partial g}{\partial E}\right|_{E_{\mathrm{ASE}}} (E - E_{\mathrm{ASE}}) + \frac{1}{2} \left.\frac{\partial^2 g}{\partial E^2}\right|_{E_{\mathrm{ASE}}} (E - E_{\mathrm{ASE}})^2 + \dots \\ &\approx g_{\max} - \tfrac{1}{2} |g''| (E - E_{\mathrm{ASE}})^2, \end{aligned} \tag{S3}$$

where g'' is the curvature of the gain spectrum at its maximum. Since the threshold pump fluence is inversely proportional to the net gain, $P_{\mathrm{th}} \propto 1/g(E)$, we obtain:

$$P_{\mathrm{th}}(\Delta E) \approx P_{\mathrm{th,\,min}} \left[1 + \left(\frac{\Delta E}{w} \right)^2 \right], \tag{S4}$$

where w is a characteristic gain width related to the curvature of the gain spectrum, and $P_{\mathrm{th,\,min}}$ is the minimum threshold at the ASE centre. This parabolic threshold dependence is directly confirmed by our independent threshold measurements (Fig. 2d of the main manuscript, left axis), which show that the lasing threshold is minimised when the spectral offset from the exciton matches the energies of the dominant vibrational modes V_2 and V_3 ($\sim$193–198 meV, identified from the Raman spectrum of MeLPPP), corresponding to the condition of most efficient vibron-mediated relaxation from the exciton reservoir to the lasing mode.

Step 2: Saturation parameter at threshold

Under non-resonant pumping at 400 nm, all intracavity molecules are excited with equal probability regardless of their coupling to the cavity mode. The fraction of excited molecules at threshold is:

$$\xi = \frac{\sigma_{\mathrm{abs}} P_{\mathrm{th}}}{\hbar\omega_{\mathrm{pump}}}, \tag{S5}$$

where σ_{abs} is the absorption cross section at the pump wavelength. Since σ_{abs} and $\hbar\omega_{\mathrm{pump}}$ are constant across all measurements (same material, same pump wavelength), we have $\xi \propto P_{\mathrm{th}}$. Substituting the parabolic threshold from Eq. (S4):

$$\xi(\Delta E) = \xi_{\min} \left[1 + \left(\frac{\Delta E}{w} \right)^2 \right]. \tag{S6}$$

Step 3: Combining contributions

From Eq. (S2), the saturation-induced blueshift is linear in ξ in the small-saturation regime ($\xi \ll 1$). Denoting the proportionality constant (which depends on $\hbar\Omega_0$, δ, n_{eff}, α, and the FWHM) as C, we have:

$$\Delta E_{\mathrm{sat}} = C \cdot \xi(\Delta E) = C \cdot \xi_{\min} \left[1 + \left(\frac{\Delta E}{w} \right)^2 \right]. \tag{S7}$$

Combining all contributions, the total energy shift is:

$$\boxed{E_{\mathrm{lasing}} - E_{k_0} = A_{\mathrm{sat}} \left(\frac{\Delta E}{w} \right)^2 + A_{\mathrm{pull}}\, \Delta E + E_0,} \tag{S8}$$

where $A_{\mathrm{sat}} = C \cdot \xi_{\min}$ is the amplitude of the threshold-dependent symmetric blueshift, A_{pull} is the gain-pulling coefficient, and $E_0 = C \cdot \xi_{\min} + \Delta E_{\mathrm{pull}}(0)$ is the baseline blueshift at the ASE centre. Each parameter is directly traceable to a physical mechanism identified in Ref. (7).

We note that the parabolic approximation is strictly valid near the gain maximum; at large spectral offsets ($|\Delta E| \gg w$), the gain profile falls off faster than a parabola (Gaussian or Lorentzian tails), leading to a threshold that increases more steeply and correspondingly larger blueshifts than the parabolic model predicts.

4.3 Quantitative analysis of the fitted parameters

The quantitative analysis reveals that the dominant contribution to the energy shift is the symmetric, threshold-dependent saturation term (A_{sat}), which is present for all cavity orders from the strongly coupled $M = 3$ to the weakly coupled $M = 17$, consistent with the conclusion of Ref. (7) that saturation-induced blueshifts operate independently of the coupling regime. The parabolic dependence on spectral offset from the ASE peak directly mirrors the independently measured parabolic dependence of the lasing threshold, which we attribute to vibron-mediated relaxation efficiency (V_2 and V_3 modes at ∼193–198 meV, as identified from the Raman spectrum of MeLPPP), establishing a quantitative, causal link between the threshold variation and the energy shift pattern via the saturation parameter ξ.

Gain-induced frequency pulling, by contrast, is negligible: the antisymmetric pulling coefficient $|A_{\mathrm{pull}}| < 0.015$ for all cavity orders, contributing less than 1 meV even at 50 meV spectral offset — a direct consequence of the narrow cavity linewidths relative to the broad ASE gain spectrum of MeLPPP, and consistent with Ref. (7).

While the V-shape itself is thus universal, the baseline blueshift E_0 differs between coupling regimes: the $M = 3$ cavity ($\hbar\Omega_0 \approx 170$ meV) exhibits $E_0 = +3.1$ meV, significantly larger than the near-zero baselines observed for $M = 8$, 9, and 17, consistent with residual Rabi quenching even at the minimum threshold excitation density — a channel that is absent in the weak-coupling regime. Thus, while the observation of blueshifts alone does not distinguish polariton from photon lasing, in full agreement with Ref. (7), the quantitative decomposition of the energy shift into its spectral-offset-dependent and baseline components reveals coupling-regime-dependent information.

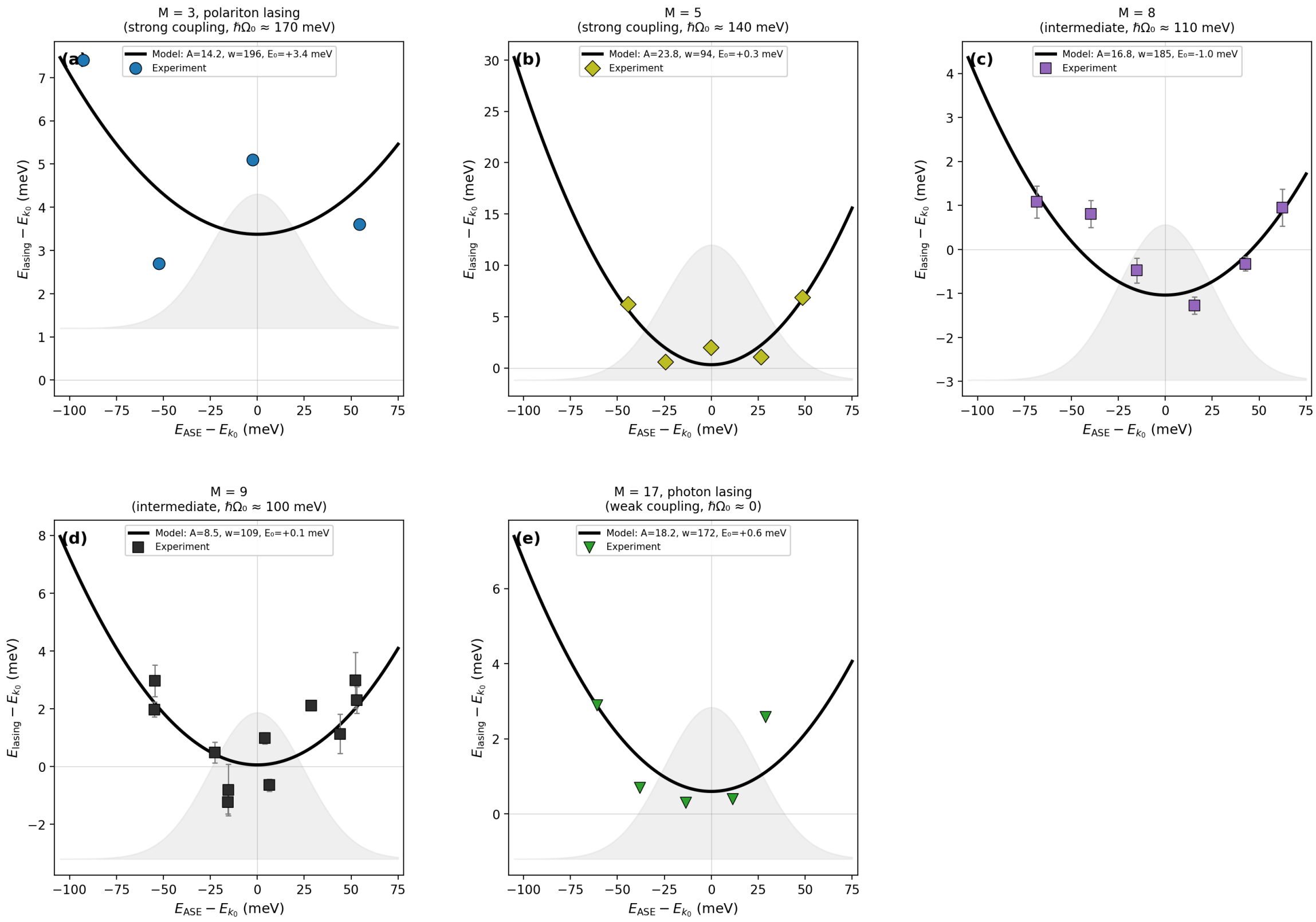

Figure S4: Parabolic V-shape fit of the nonlinear energy shift across five cavity-mode orders. The nonlinear energy shift at $\approx 2 \times P_{\mathrm{th}}$ relative to the below-threshold emission at $k_{||} = 0$, $E_{\mathrm{lasing}} - E_{k_0}$, is plotted as a function of the spectral offset from the ASE maximum, $\Delta E = E_{\mathrm{ASE}} - E_{k_0}$, for **a**, $M = 3$; **b**, $M = 5$; **c**, $M = 8$; **d**, $M = 9$; and **e**, $M = 17$. Black solid curves show best fits to the parabolic V-shape model, $E_{\mathrm{lasing}} - E_{k_0} = A_{\mathrm{sat}}(\Delta E/w)^2 + E_0$ (antisymmetric component fixed at $A_{\mathrm{pull}} = 0$; see Section 4). Fitted parameters are given in each legend. The grey shaded region shows the normalised ASE spectrum of a 180-nm MeLPPP reference film, plotted for visual reference. Error bars, where shown, represent the standard deviation of repeated threshold measurements.

5 Optical Setup for Strong- and Weak-coupling Characterization

All real- and momentum-space imaging experiments described in the main text were performed using the following experimental scheme. The sample was mounted on an XYZ stage at the focal plane of the focusing objective. A beam sampler before the sample was used to direct a portion of the pump beam to a Thorlabs DET 10A photodetector connected to an oscilloscope (Picoscope 2000). The pump pulse energy was controlled using a gradient ring ND filter. Data acquisition was automated via a custom LabView (National Instruments) program, operating in a time-integrated mode below the lasing threshold and a single-shot mode above the lasing threshold. The single-shot regime allowed for the synchronous acquisition of individual pump pulse amplitudes and their corresponding photoluminescence emissions, thus preventing averaging over the intensity fluctuations of the laser. For measuring reflectivity spectrum of the microcavity an Ocean Optics DH-2000 fiber-coupled Halogen–Deuterium white light source was

used.

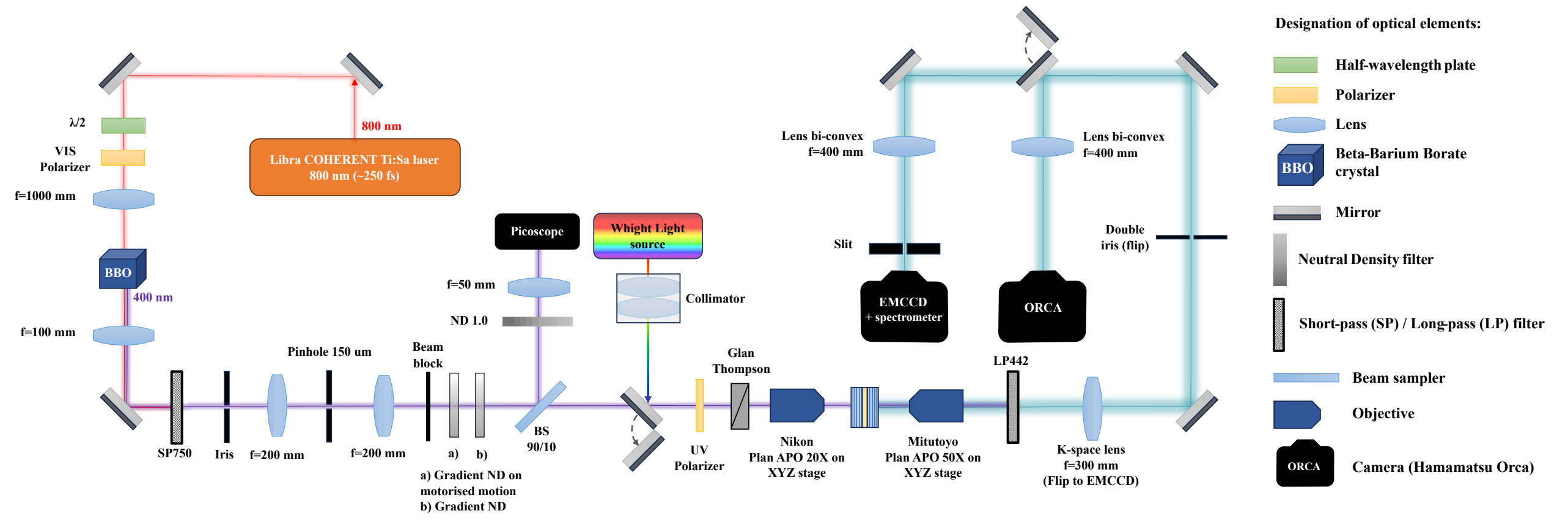

Figure S5: Schematic of the experimental setup for optical characterisation of photoluminescence emission from the investigated microcavity.

References

[1] R. Ammenhäuser, A. Helfer, and U. Scherf, "Reliably estimating the length of the effectively conjugated segment in ladder poly(para-phenylene)s," *Org. Mater.*, vol. 02, pp. 159–164, Apr. 2020.

[2] T. Zhai, K. Ambrose, A. Nyayachavadi, K. G. Walter, S. Rondeau-Gagné, and J. I. Feldblyum, "Ferrocene metallopolymers of intrinsic microporosity (MPIMs)," *Chem. Commun. (Camb.)*, vol. 58, pp. 238–241, Dec. 2021.

[3] A. G. Bonn, O. Yushchenko, E. Vauthey, and O. S. Wenger, "Photoinduced electron transfer in an anthraquinone-$[Ru(bpy)_3]^{2+}$-oligotriarylamine-$[Ru(bpy)_3]^{2+}$-anthraquinone pentad," *Inorg. Chem.*, vol. 55, pp. 2894–2899, Mar. 2016.

[4] U. Scherf and K. Müllen, "Polyarylenes and poly(arylenevinylenes), 7. a soluble ladder polymer via bridging of functionalized poly(*p*-phenylene)-precursors," *Makromol. Chem.*, vol. 12, pp. 489–497, Aug. 1991.

[5] "PREPARATION OF LOW-HALIDE METHYLLITHIUM," *Organic Synth.*, vol. 62, p. 101, 1984.

[6] U. Scherf, A. Bohnen, and K. Müllen, "Polyarylenes and poly(arylenevinylene)s, 9 the oxidized states of a (1,4-phenylene) ladder polymer," *Makromol. Chem.*, vol. 193, pp. 1127–1133, May 1992.

[7] T. Yagafarov, D. Sannikov, A. Zasedatelev, K. Georgiou, A. Baranikov, O. Kyriienko, I. Shelykh, L. Gai, Z. Shen, D. Lidzey, and P. Lagoudakis, "Mechanisms of blueshifts in organic polariton condensates," *Communications Physics*, vol. 3, p. 18, Jan 2020.
